# Method of Using Inlay Gold in Making an Open Face Crown

**Published:** 1907-08

**Authors:** W. H. Hoyl

**Affiliations:** Dawson, Ga.


### METHOD OF USING INLAY GOLD IN MAKING AN OPEN FACE CROWN.

By W. H. Hoyl, D. D. S., Dawson, Ga.

I believe we all agree in saying there are cases when the open face crown is clearly indicated, the great hindrance being in making a perfect adaptation. Follow the details of this method carefully, and I believe you will be pleased with the result.

Take the impression of the tooth to be crowned in plaster, producing a plaster model; trim model at cervical margin carefully with view of having finished crown extend under free margin of gum. Make impression in mouldine and run model in Melotte's metal. Polish metal model with cuttlefish disk. Take inlay gold (preferably 24 k. one one-thousandth), cut a piece sufficiently large to cover the labial, and one for the lingual surfaces, cutting it large enough for overlap of one thirty-second of an inch in interdental spaces. With the assistance of slightly softened modeling composition you can readily approximately adapt this inlay gold to the metal model. For more perfect swaging, place the two sides of the crown in position on the metal model, and over this place the metal ring which comes with all mouldine outfits, and it being of same size rests firmly upon edges of model. Fill the ring flush with wet cotton and pack it down with a wooden piston (home made), tapping it lightly with an ordinary hammer. Remove cotton and you will find the two sides of the crown stuck together, and they can be removed from metal model without disturbing this relation. Now place the perfectly swaged crown on asbestos mat, and with a small piece of 22 k. solder unite the two sides. Place on plaster model and cut out face with carborundum stone. Try on natural tooth and with oval burnisher adapt margins as you would in case of making an inlay filling. We now have a perfect matrix of the tooth to be crowned. Invest it by means of filling the crown, or matrix, with investment material, either fine marble dust or sump, bringing to a heat, and flow 22 k. solder over the entire surface. Finish just as you would an inlay filling.—Items of Interest.

